# Probiotic Bacteria for Healthier Aging: Immunomodulation and Metabolism of Phytoestrogens

**DOI:** 10.1155/2017/5939818

**Published:** 2017-10-01

**Authors:** José María Landete, Pilar Gaya, Eva Rodríguez, Susana Langa, Ángela Peirotén, Margarita Medina, Juan L. Arqués

**Affiliations:** Departamento Tecnología de Alimentos, Instituto Nacional de Investigación y Tecnología Agraria y Alimentaria (INIA), Carretera de La Coruña Km 7, 28040 Madrid, Spain

## Abstract

Age-related degeneration gives rise to a number of pathologies, many of them associated with imbalances of the microbiota and the gut-associated immune system. Thus, the intestine is considered a key target organ to improve the quality of life in senescence. Gut microbiota can have a powerful impact in the deterioration linked to aging by its nutritional and immunomodulatory activity. Reduced numbers of beneficial species and low microbial biodiversity in the elderly have been linked with pathogenesis of many diseases. A healthy lifestyle with an elderly customized diet including probiotics can contribute to reducing the chronic proinflammatory status and other age-related pathologies. Beneficial effects of probiotic lactic acid bacteria and bifidobacteria to alleviate some of these disorders based on their immunomodulatory properties as well as their capacity to produce bioactive metabolites from dietary phytoestrogens are summarized. On one hand, the preservation of gut barrier integrity and an increased ability to fight infections are the main reported immune benefits of probiotics. On the other hand, the intake of a diet rich in phytoestrogens along with the presence of selected probiotic bacteria may lead to the production of equol, enterolignans, and urolithins, which are considered protective against chronic diseases related to aging.

## 1. The Aging Process

The time-dependent biological complex processes that produce a gradual generalized deterioration of the anatomy and physiological functions of organisms are defined as aging. It led to weakness to environmental stress and therefore increases the risk of disease and death. Among multicellular organisms, aging is marked by a progressive decline in the function of multiple cells and tissues. Apparently, the event of aging is genetically determined and modulated by the environment, but the causes of those irreversible changes are still an unresolved challenge. Understanding aging is an important objective that may help to modify the aging process or the senescence effects. The aging rate could be determined by two major circumstances: the accumulation of damage and the effectiveness of somatic maintenance mechanisms [1]. Nine cellular and molecular hallmarks of aging have been proposed by López-Otín et al. [2], which are genomic instability, telomere attrition, epigenetic alterations, loss of proteostasis, deregulated nutrient sensing, mitochondrial dysfunction, cellular senescence, stem cell exhaustion, and altered intercellular communication. In human cells, the presence of telomerase suggests that cells may be programmed to undergo senescence as a mechanism to “count” cell divisions, although stress and damage accumulation are also important for the telomere shortening [3].

The main aim of aging research is to improve the quality of life. Age-related degeneration gives rise to a number of pathologies, such as osteoarthritis, atherosclerosis, lung emphysema, malignancies (gastrointestinal, prostate), and dementias. The aging process is dependent on antistress responses, which act as antiaging mechanisms. Furthermore, immunosenescence, which can be defined as a decline in the functionality of the immune system, contribute to a chronic state of basal inflammatory activity (inflammaging) [4–6].

The most studied and reproducible nongenetic intervention in aging research is dietary restriction. However, the importance of diet composition has been highlighted when applying a reduction in calorie intake to regulate the lifespan [7]. Another important factor that can play a key role in senescence is the impact of the diet on the gut microbiota composition and the chronic inflammation. Thus, age-related changes in the nutritional behaviour are associated with the imbalances of the microbiota and the gut-associated immune system. A healthy lifestyle with an elderly customized diet including probiotics can contribute to reduce the chronic proinflammatory status and other age-related pathologies [8–10].

### 1.1. Aging and Gut

The gastrointestinal (GI) tract is characterized by its complexity, being the main and largest site for interaction with the external environment. The GI tract is covered by a single layer of epithelial cells, which are responsible for the digestion and absorption of nutrients and electrolytes, as well as homeostasis. Moreover, the gut-associated lymphoid tissue provides an important first line of defence that controls the equilibrium between tolerance and immunity against orally acquired food and microbes. The human gut contains the enteric microbiota, whose mutualistic relationship contributes to the maintenance of health, including digestion of complex carbohydrates, intestinal homeostasis, synthesis of essential nutrients and vitamins, protection against pathogens, and stimulation of the immune system [11]. Age-associated modifications of the gut cause disorders that clearly affect the quality of life of elderly population, becoming a major cause of morbidity [12].

A distinguishing characteristic of the aging gut is the overexpression of proinflammatory cytokine IL-6, which has an effect on the intestinal barrier function and mucosal immune system [13]. Persistence of inflammaging can also facilitate cancer development and progression [6, 14]. During postmenopause/andropause periods IL-6 levels are increased. Overexpression of IL-6 might have important ramifications with regard to both impaired immunity and intestinal barrier integrity, which can downregulate innate immunity to pathogens and consequently increase the susceptibility to infections in the elderly. Moreover, those changes in the intestinal permeability could be crucial in the development of local (celiac disease, colorectal cancer, or inflammatory bowel disease) and systemic diseases (diabetes, chronic heart failure, or obesity) and even in central nervous system disorders [15, 16].

Physical and immunological impairments of intestinal barrier are correlated with age-related diseases and lifespan. The cross-talk between gut microbiota and the gut-associated lymphoid tissue has a powerful effect on the host immune response which can lead to systemic metabolic effects [17]. Thus, the intestine is a key target organ to improve the quality of life in senescence [18, 19].

### 1.2. Impact of Gut Microbiota on Aging

Alterations in morphology and physiological functions modify the physical environment of the elderly gut, which affect the composition of the intestinal microbiota. Moreover, antibiotics are still an irreplaceable therapy for the elderly, which have also a huge influence on the intestinal microbiota composition. Dysbiosis is associated with various metabolic, infectious, and inflammatory disorders including malnutrition, diabetes, bowel diseases,* Clostridium difficile* infections, obesity, colon cancer, and atherosclerosis [20, 21]. An interesting clue to unravel the role of gut microbiota in some aged-related diseases is the big interindividual variations among older subjects compared to the adults [8, 22].

Gut microbiota has a strong impact in human physiology and, therefore, on the health status in the elderly and age-related diseases [23]. Its immunomodulatory properties could help in two main aspects of aging as immunosenescence and inflammaging. Aging can be considered as an immune disorder [24]. Commensal bacteria can modulate the host inflammatory response, mainly by targeting NF-*κ*B. It has been proposed that an increased presence of IL-6-inducing bacteria in the elderly could be associated with elevated intestinal levels of IL-6 in the gut and therefore at systemic level [14]. Thus, an aged-type microbiota shows a low microbial biodiversity, enriched in pathobionts and facultative anaerobes and depleted of* Firmicutes*, which is linked with an increase of proinflammatory signals [22, 25–27]. Another important aspect to address during the aging process is the interaction between the microbiota and the metabolism of dietary components and their potential beneficial effects in the generation of bioactive nutrients [28, 29].

Host age, health status, and environmental factors can modulate our microbiota composition. Improving the profile of the gut microbiota during human aging, mainly lifestyle factors and nutritional habits, would have an impact on human health and longevity since longevity process is associated with human gut microbiota changes [30]. The role of gut microbiota in human aging include two main aspects: immunomodulatory and nutritional (energy availability and metabolism). Dietary interventions with probiotics or fecal bacteriotherapy could be employed to rationally enrich the gut microbiota of the elderly [20, 30–33].

## 2. Potential Beneficial Effects of Probiotics on the Aging

Probiotics can be applied to modulate the age-related gut microbiota imbalance and to introduce strains with specific health-promoting effects. The principal claimed benefits of probiotics in elderly people are prevention of diarrheal diseases, protection against pathogens, enhancement of the intestinal barrier function, improvement of gastrointestinal motility and inflammatory intestinal disorders, immunomodulatory effects, and prevention of colon cancer [34, 35].

Probiotic intervention, with or without a specific diet composition, would help to improve the microbiota functionality in order to obtain health benefits during the old age. In this context, a diet rich in phytoestrogens can be considered an interesting therapeutic approach against aging due to their estrogenic and antioxidant actions. Here we summarize two promising beneficial effects of probiotics to alleviate some age-related pathologies based on their immunomodulatory properties as well as their capacity to produce bioactive metabolites from dietary compounds, such us phytoestrogens.

### 2.1. Probiotics to Improve Immune-Health

Senescence is associated with a decline in immune function and an increase in inflammation [10]. The effects of IL-6 on intestinal permeability could increase the penetration of microbes and/or toxins into the body [10, 36]. Probiotic intervention can improve some of these age-associated modifications of the immunological features [37–39]. However, despite their promising benefits, little is known about the effect probiotics on intestinal barrier and immune function.

Probiotics can exert beneficial effects on the preservation of gut barrier integrity and function stimulating the activity and growth of beneficial bacteria and regulating the expression of tight junction proteins [40–47].

Aging process affects innate immunity, with reduced activity or number of natural killer (NK) cells, and adaptive immunity, with reduced antigen-specific IgA antibody and cellular immune responses [48]. Probiotic treatments can ameliorate some of these processes modulating cytokine production, improving distribution and function of NK cells, macrophages, granulocytes, and T cells in the circulation, and enhancing mucosal and systemic antibody responses [49–51].

Lactic acid bacteria (LAB) and bifidobacteria are commonly found in the gut of humans and other animals as well as in probiotic supplements and foods. Their immunomodulatory properties can be applied in age-related disorders. Studies carried out on mice demonstrated the potential of probiotics to palliate the effects of aging on the immune system. Administration of* Lactococcus lactis* H61 or* L. rhamnosus* MTCC 5897 improved the age-associated Th1/Th2 imbalance [52, 53].* Bifidobacterium adolescentis* BBMN23 and* Bifidobacterium longum* BBMN68 isolated from healthy centenarians enhanced both innate and acquired immunity in mice [54]. Supplementation of aged mice with the probiotic* Lactobacillus paracasei* NCC2461 improved the specific adaptive immune response, with higher IgG2a levels after antigenic challenge [55]. The strain* L. rhamnosus* CRL1505 was able to increase the peritoneal macrophages phagocytic activity and the number of intestinal IgA^+^ cells in the intestinal mucosa of aged mice [56]. Recently, the effect of* Lactobacillus plantarum* WCFS1,* L. casei* BL23, and* Bifidobacterium breve* DSM20213 on gut barrier and immunity in accelerated aging mice was investigated. That study found that age-related decline in mucus and systemic immunity can be modulated by probiotics but also highlights the risk of translating the beneficial effects of probiotics observed in young animals or humans to the elderly [57].

Several human studies also show a higher ability to fight infections following probiotic consumption.* Bifidobacterium lactis* HN019 enhanced phagocytic activity and number of NK cells in elderly subjects [51, 58, 59]. A probiotic cheese containing* Lactobacillus rhamnosus* HN001 and* Lactobacillus acidophilus* NSFM increased the cytotoxicity of NK cells in elderly volunteers [60]. Administration of yogurt containing the probiotic strain* Lactobacillus casei* DN-114001 to elderly people reduced the length of winter infections compared to the control group [61]. Likewise, an improvement in the nutritional and immunological status of enterally fed elderly subjects was observed by the administration of a fermented milk containing* Lactobacillus johnsonii *La1 [62].

### 2.2. Probiotics, Phytoestrogens, and Aging

Phytoestrogens are polyphenols present in plants or foods derived from plants foods such as soya, flaxseed, cereals, vegetables, fruit, chocolate, and tea [63–65]. Phytoestrogens such as coumestans, stilbenes, ellagitannins, lignans, and isoflavones are similar to endogenous estrogen and therefore they have both antiestrogenic and estrogenic effects [66]. Intake of these compounds may be protective against chronic diseases related to aging, such as cardiovascular and bone diseases, various cancers, menopausal symptoms, and cognitive function [67–73]. These health benefits from phytoestrogens consumption should be attributed to the bioactive metabolites produced by gut bacteria and to the modulation of the intestinal bacterial population [74, 75]. Thus, the intake of a diet rich in isoflavones (soybeans and soy derived foods), lignans (flax seeds, cereals, etc.), and/or ellagitannins (pomegranates, cherries, etc.) along with the presence of selected probiotic bacteria may ensure the production of equol, enterolignans, and urolithins in the gut, respectively [76–78] (Table 1). This approach should be considered in the prevention and improvement of aging-related pathologies.

The transformation of isoflavones, lignans, and ellagitannins by bacteria is an essential step because

**Figure 1:**
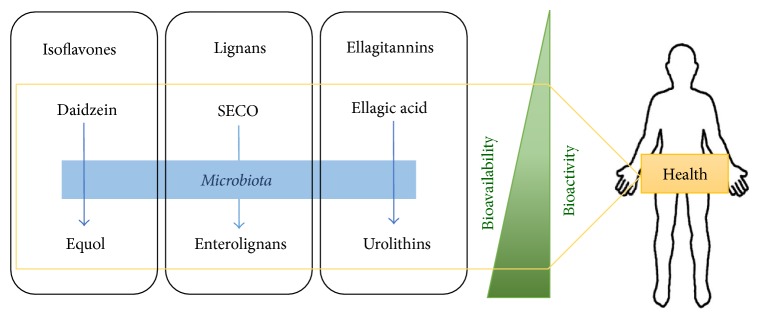
Isoflavones, lignans, and ellagitannins intake are metabolized by potential probiotic bacteria to produce equol, enterolignans, and urolithins, respectively. These compounds are more bioavailable and bioactive than their precursors.

1. equol, enterolignans, and urolithins are more bioavailable than their respective dietary phytoestrogens [79, 80] (Figure 1),
2. equol, enterolignans, and urolithins have more estrogenic/antiestrogenic activities than their precursors. The biological action of these derived compounds is mediated primarily by estrogen receptors [81], modulating hormone levels and expression of estrogen receptors [82, 83]. They may act as anticarcinogens through antiestrogenic actions competing with estradiol to bind estrogen receptors [84]. Equol, enterolignans, and urolithins have various estrogenic effects in postmenopausal women, such as decreased plasma levels of estrone and estradiol sulfate and changes in the metabolism of estrogen (from 16*α*-hydroxylation to 2-hydroxylation, a less carcinogenic pathway) [85, 86],
3. equol and enterolignans are more antioxidants than their precursors [80, 87], acting against DNA damage and lipid peroxidation. The antioxidant activities of enterolignans have also been suggested to contribute to the reduction of hypercholesterolemia, hyperglycemia, and atherosclerosis [88],
4. finally, equol, enterolignans, and urolithins have anti-inflammatory effects and exert antiproliferative and apoptosis-inducing activities [89, 90].

Although specific bacteria responsible for the equol, enterolignans, and urolithin production are still being investigated, some LAB and bifidobacteria have been involved in the metabolism of these compounds [78, 91].

#### 2.2.1. Isoflavones, Aging, and Probiotic Bacteria

In soy and unfermented soy foods, isoflavones are as glycosides such as daidzin, genistin, or glycitin. These compounds are less estrogenic than their aglycones daidzein, genistein, and glycitein, respectively. Daidzin, genistin, or glycitin cannot be absorbed because of their higher molecular weights and hydrophilicity [92]. Then, their bioavailability requires the transformation in daidzein, genistein, and glycitein by means of *β*-glycosidase activities.

Benefits of soy in aging are derived from the isoflavones metabolism of bacteria, including protection against breast cancer [93], prostate cancer [94], menopausal symptoms [95], heart disease [96], osteoporosis [97], and cognitive function [98].

LAB and bifidobacteria are very important in the transformation of naturally occurring isoflavones in the form of* O*-glucosides,* C*-glucosides, or their methylated forms in the bioactive isoflavones daidzein and genistein and even in the formation of dihydrodaidzein [91]. The capabilities of converting daidzin to daidzein have been observed in* Weissella confusa*,* Enterococcus durans* KH, and* Lactobacillus paraplantarum* KM [99], as well as in* L. rhamnosus* CRL981 [100].* Lactobacillus* sp. Niu-O16, isolated from bovine rumen contents, converted daidzein to dihydrodaidzein [101].

Daidzein, genistein, dihydrodaidzein, and dihydrogenistein possess physiological properties of interest in healthy aging [68]. The production of daidzein and dihydrodaidzein facilitates the formation of equol and/or* O*-desmethylangolensin (*O*-DMA). Equol has enhanced effects due to its greater affinity for estrogen receptors, unique antiandrogenic properties, and superior antioxidant activity.* In vivo* and* in vitro* beneficial effects of equol have been demonstrated [102]. So, it has been possible to demonstrate* in vitro* the effect of equol against aging in skin [103] and nervous system [104]. On the other hand, the effect of equol in the improvement of menopause symptoms and in the prevention of cancers and cardiovascular diseases has been demonstrated both* in vitro* [105] and* in vivo* [106–108]. Evidence from* in vitro* studies suggests that* O*-DMA may have several cancer-related biological actions. However, results from human metabolic studies and observational studies of disease risk suggest that these actions may not be physiologically relevant* in vivo* due to the amount and form (primarily glucuronide) of circulating* O*-DMA [109].

A mix of bacteria composed of* Finegoldia magna* EPI3,* Lactobacillus mucosae *EPI2,* Enterococcus faecium* EPI1, and* Veillonella* sp. strain EP was able to transform daidzein into equol [110]. Similarly, anaerobic incubation of* Eggerthella* sp. Julong 732 and* Lactobacillus *sp. Niu-O16 transformed dihydrodaidzein to S-equol [111], although most of equol-producing microorganisms belonging to the Coriobacteriaceae family,* Lactococcus garvieae* 20–92 [112],* B. breve* 15700, and* B. longum* BB536, were also able to produce equol [113]. LAB and bifidobacteria are also indirectly involved in the production of equol, facilitating the formation of precursor metabolites or favoring the presence of equol-producing bacteria. The administration of* Lactobacillus gasseri* influences the effect of isoflavonoids on the host, probably through changes in the gastrointestinal environment [114].

#### 2.2.2. Lignans, Aging, and Probiotic Bacteria

Lignans, which are the major phytoestrogens occurring in Western diets, have relevant health properties [115]. However, plant lignans are not usually absorbed and must be metabolized to enterodiol and enterolactone prior to absorption [67, 116]. These compounds are the main responsible agents for the beneficial effects of lignans [117]. The transformation of plant lignans by intestinal microbiota is essential for the manifestation of these functions [118]. Enterolignans could be used in ameliorating some menopausal symptoms, protecting against atherosclerotic plaque deposition and due to their hepatoprotective effects [119–122].

Deglycosylation of the secoisolariciresinol diglucoside (SDG) present in the lignan extracts into secoisolariciresinol (SECO) is the first step towards the formation of enterolignans. The production of SECO from lignan extracts and SDG is widespread within LAB and bifidobacteria isolates [78, 123]. SDG hydrolysis is an important feature in probiotic bacteria to enhance the release of SECO, improving its bioavailability for absorption by colonic mucosa and/or the biotransformation to enterodiol and enterolactone by intestinal microorganisms [118, 124].

Nowadays, different bacteria such as* Butyribacterium methylotrophicum*,* Eubacterium callanderi*, and* Peptostreptococcus productus* and the strains* Eubacterium limosum*,* Ruminococcus productus*,* Clostridium scindens, Peptostreptococcus productus* SECO-Mt75m3, and* Eggerthella lenta* SECO-Mt75m2 have been involved in the production of enterolignans [65, 118]. Recently, we have described the first probiotic bacterium (*B. adolescentis* INIA P784) capable of metabolizing lignan extracts to produce enterodiol, being the first time that the production of enterolignans by a unique bacterium strain is registered [78].

#### 2.2.3. Ellagitannins, Aging, and Probiotic Bacteria

Ellagitannins are complex derivatives of ellagic acid, which are largely metabolized by the colon microbiota of different mammals [125, 126] and humans prior to absorption [127, 128]. The microbially mediated origin of urolithin has been demonstrated [129, 130]. Ellagitannins, ellagic acid, and urolithins exhibit anticancer properties* in vitro* and* in vivo* [69, 131]. Pomegranate extracts inhibit the growth of lung, prostate, colon, and breast cancer cells* in vitro* [132–135]. Urolithins inhibit mitogen-activated protein kinase signalling [136], which could curtail the risk of development of colon cancer by inhibiting cell proliferation and inducing apoptosis [90].

To date, only two urolithin-producing strains,* Gordonibacter urolithinfaciens* CEBAS 1/15P and* Gordonibacter pamelaeae* DSM 19378, have been identified [137, 138]. However, these strains cannot produce the downstream products urolithin A and urolithin B. Unraveling the bacterial phyla or group of bacteria responsible for production of these compounds is of great interest since they can be potentially used as probiotics [139]. Consumption of foods containing ellagic acid is also associated with health beneficial effects, and they could be mediated by the presence of urolithin-producing microorganisms [77].

Probiotics able to produce or to increase species related to the production of urolithins or other phytoestrogens such as equol and enterolignans can mean a step forward in the probiotic interventions, increasing the bioavailability of these compounds, and subsequently their therapeutic applications.

## 3. Conclusion

Age-related changes in nutritional behaviour and microbial diversity during aging result in a higher susceptibility to infections and diseases. Likewise, the presence of some beneficial microorganisms in the gut could help to prevent or delay some age-associated diseases by improving the immune response, or by the production of bioactive metabolites as equol, enterolignans, and urolithins. The evidence for intake of probiotics along with age specifically oriented diet to improve the health during aging is promising. However, further studies for a rational manipulation of the gut microbiota are needed to better define the role of probiotics and to assess the real potential of these interventions.

## Figures and Tables

**Table 1:** Potential probiotic strains implicated in the metabolism of phytoestrogen.

Bacteria	Transformation/production	Reference
*Lb. rhamnosus* CRL981	Daidzin to daidzein	[100]
*Lb. plantarum* CECT 748T	Daidzin to daidzein	[80]
*Lactobacillus* sp. Niu-O16	Daidzein to dihydrodaidzein	[101]
*Lb. rhamnosus* INIA P540	Daidzin to dihydrodaidzein	[91]
*Ent. faecalis* INIA P333	Daidzin to dihydrodaidzein	[91]
*Lb. mucosae* EPI2, *Ent. faecium* EPI1, *Finegoldia magna* EPI3, and *Veillonella* sp. EP	Daidzein into equol	[110]
*Lactococcus garvieae* 20–92	Daidzein into equol	[112]
*B. breve* 15700 and *B. longum* BB536	Daidzein into equol	[113]
*B. adolescentis* INIA P784	Enterodiol production from flax seed	[78]
*Gordonibacter urolithinfaciens* and *Gordonibacter pamelaeae* DSM 19378T	Urolithin C from ellagic acid	[137]
